# Cortico-Cortical Interactions during Acquisition and Use of a Neuroprosthetic Skill

**DOI:** 10.1371/journal.pcbi.1004931

**Published:** 2016-08-19

**Authors:** Jeremiah D. Wander, Devapratim Sarma, Lise A. Johnson, Eberhard E. Fetz, Rajesh P. N. Rao, Jeffrey G. Ojemann, Felix Darvas

**Affiliations:** 1 Department of Bioengineering, University of Washington, Seattle, Washington, United States of America; 2 Department of Neurological Surgery, University of Washington, Seattle, Washington, United States of America; 3 Department of Physiology and Biophysics, University of Washington, Seattle, Washington, United States of America; 4 Department of Computer Science and Engineering, University of Washington, Seattle, Washington, United States of America; Indiana University, UNITED STATES

## Abstract

A motor cortex-based brain-computer interface (BCI) creates a novel real world output directly from cortical activity. Use of a BCI has been demonstrated to be a learned skill that involves recruitment of neural populations that are directly linked to BCI control as well as those that are not. The nature of interactions between these populations, however, remains largely unknown. Here, we employed a data-driven approach to assess the interaction between both local and remote cortical areas during the use of an electrocorticographic BCI, a method which allows direct sampling of cortical surface potentials. Comparing the area controlling the BCI with remote areas, we evaluated relationships between the amplitude envelopes of band limited powers as well as non-linear phase-phase interactions. We found amplitude-amplitude interactions in the high gamma (HG, 70–150 Hz) range that were primarily located in the posterior portion of the frontal lobe, near the controlling site, and non-linear phase-phase interactions involving multiple frequencies (cross-frequency coupling between 8–11 Hz and 70–90 Hz) taking place over larger cortical distances. Further, strength of the amplitude-amplitude interactions decreased with time, whereas the phase-phase interactions did not. These findings suggest multiple modes of cortical communication taking place during BCI use that are specialized for function and depend on interaction distance.

## Author Summary

The neurons in the human brain are densely interlaced, sharing upwards of 100 trillion physical connections. It is widely theorized that this tremendous connectivity is one of the facets of our nervous system that enables human intelligence. In this study, over the course of a week, human subjects learned to use electrical activity recorded directly from the surface of their brain to control a computer cursor. This provided us an opportunity to investigate patterns of interactivity that occur in the brain during the development of a new skill. We demonstrated two fundamentally different forms of interactions, one spanning only neighboring populations of neurons and the other covering much longer distances across the brain. The short-distance interaction type was notably stronger during early phases of learning, lessening with time, whereas the other was not. These findings point to evidence of multiple different forms of task-relevant communication taking place between regions in the human brain, and serve as a building block in our efforts to better understand human intelligence.

## Introduction

Direct communication between brain and machine provides a powerful platform for both the development of clinical therapies and scientific inquiry. By providing the brain with a completely novel output pathway, experimentalists have an opportunity to observe the ways in which the brain responds to and develops control over this new output mechanism [1]. A number of studies have demonstrated that the use of a brain-computer interface (BCI) is a learned skill [2–6], and that the brain can learn this skill more effectively when the transformation that maps neural activity to BCI control is consistent [7]. Further, it has been demonstrated that the nature of the neural signals being used to drive the BCI changes with practice [4,8] and that there are also changes in neural activity in populations that are not directly linked to BCI control [both local to the controlling site [9]; and at more remote sites [8]]. The mechanisms underlying learning of BCI control have many similarities to those for learning motor control [10]. Repeated BCI training can have lasting effects on motor networks, altering functional connectivity in cortico-thalamic networks during execution of a finger-tapping task [11]. To date, there have been no systematic studies of cortico-cortical interaction during BCI use. Other than a recent study demonstrating the need for corticostriatal interaction during the BCI learning process in a rodent model [12], we have little understanding of the networks involved in acquisition of the neuroprosthetic skill.

Electrophysiological signals for BCI control can be derived at a variety of spatial scales, from single unit recordings to surface electroencephalography [13]. Field potentials contain a number of features that have been demonstrated to hold neurophysiological relevance to motor function: mu (8–12 Hz), beta (15–31 Hz), and high gamma (HG, 70–150 Hz). HG activity is considered a marker of local cortical activity [14] and is positively correlated with motor activity [15], whereas mu and beta oscillations are negatively correlated with movement onset [16]. Electrocorticography (ECoG) strikes a compromise between broad coverage of multiple cortical areas and resolution of multiple spectral features of interest (sub-millisecond temporal resolution) and is thus well suited for the investigation of distributed cortical interactions.

The brain is a vastly distributed and parallelized system, requiring effective and efficient communication between both neighboring and distant neural populations [17]. Correspondingly, of equal interest to within-region changes in synchrony of neural activity are changes in interactivity between regions. Cortical, cortico-subcortical, and cortico-muscular coherence have all been observed in the mu range (8–12 Hz) during slow movements [18]. Similar observations have been made regarding long-distance synchrony in the beta range, both in cortico-cortical interactions between primary motor cortex (M1), primary somatosensory cortex and posterior parietal cortex during a visual discrimination task [19]. Another form of phase-phase synchrony, the phase locking value [20] has been used to quantify linear interactions during execution of a cognitive task [21]. While these examples are restricted to within-frequency phase-phase interactions, it has been suggested that cross-frequency (i.e. non-linear) interactions could reflect much richer cortical interactivity [22].

Interaction between neurons and neural populations encompasses a variety of neural mechanisms, including coordinated increases in firing rates, periodic synchrony, and complex feedback loops [see 23 for review]. The link between these mechanisms and their corresponding signatures in population-based physiological signals is incompletely understood. Each of these mechanisms may manifest differently in population-scale neural recordings as anything from changes in raw covariances to detectable differences in the complex non-linear coupling of spectral components. It has been hypothesized that long-distance cortical communication is mediated by a relatively small number of direct connections, because direct connection of all communicating cells across these long distances would be biologically infeasible [24,25]. Further, Buzsáki and colleagues argued that oscillatory activity is central to the maintenance of efficient cortical information flow within increasingly large and complex mammalian cortices [17]. Such a network model would be well served by the use of oscillatory synchrony, or rhythmic interactions, to allow for maximal efficiency in processing [26].

However, there are various ways in which cortical field potentials can be related. Whether these different relationships play differing roles in cortical processing, or whether they are indicative of a single underlying network of connectivity, remains an open question. It has recently been shown that though high-frequency, amplitude-amplitude correlations in cortical field potentials are predictive of underlying local structural connectivity, this relationship deteriorates over longer distances [27]. Coupling this with the theory that oscillatory synchrony is critical to long-range cortical communication leads to a testable hypothesis of distance-specificity by interaction type: when observing simultaneous amplitude-amplitude and phase-phase interactions taking place during a cognitive task such as BCI use, the former will be observed over shorter distances and the latter over longer ones.

In a previous report [8], we demonstrated frontal and parietal regions that were active during the initial use of BCI using ECoG signals from motor cortex. These areas became less active with repeated use. Here, we examine the interactions between areas outside of the site used for BCI control with reference to the signal from the controlling electrode. We hypothesized that there exist task-driven amplitude-amplitude and phase-phase interactions observable in the ECoG field potential between the site containing the controlling electrode and remote cortical structures and that these two interaction types are present on differing spatial scales.

## Materials and Methods

### Subjects

The study presented in this manuscript was a retrospective, exploratory analysis of previously collected ECoG data. To determine which subjects were eligible for inclusion in this study, the following criteria were applied: (a) subjects needed to have participated in the one-dimensional, right-justified box task; and (b) subjects needed to perform the task above chance levels in order to demonstrate intentional control. Of the 11 subjects originally eligible per these inclusion criteria, one subject was eliminated from this study based on extreme cortical distortion due to a previously resected peri-central cavernous malformation.

The remaining ten human subjects (1 female, mean age 26.9y) were all patients with intractable epilepsy who were implanted with platinum sub-dural ECoG grids (AdTech, Racine, WI) for the clinical purpose of seizure focus localization and resection. These subjects were monitored for between four and ten days before removal of the arrays and surgical resection of the seizure focus. During this time the subjects participated in multiple recording sessions, separated over one to three days. All procedures were carried out within the University of Washington Regional Epilepsy Center, either at Harborview Medical Center or Seattle Children’s Hospital after informed consent was obtained. For children under age 18 parental consent was obtained along with consent from the child (age 14 or above) or assent of the child (age 7–13). The protocol was approved by the Institutional Review Board at both institutes. Individual patient demographic information can be found in Table 1.

**Table 1:** Subjects performing BCI task. Abbreviations: right (R), left (L), frontal (F), parietal (P), temporal (T), occipital (O). Entries in the BCI type column refer to whether the subject was performing motor imagery of the tongue or hand.

SID	Gender	Age	BCI type	Coverage	Focus location
S1	M	29	Tongue	R-F/T	R posterior T/O
S2	M	27	Tongue	R-F/P/T	R F
S3	M	14	Tongue	L-F/T	L F
S4	M	22	Tongue	R-F/P/T	R mesial T
S5	F	26	Tongue	R-F/P/T	R F
S6	M	54	Hand	L-T	L T
S7	M	11	Hand	L-F	L anterior F
S8	M	29	Hand	R-F/P/T	R F
S9	M	19	Hand	R-T	R mesial T
S10	M	38	Tongue	R-F/T	bilateral—no resection

The physical makeup (number and arrangement of electrodes) and implant location of all grids were based on clinical indication. Arrays were either 8x8, 6x8, 4x8, or 2x8 grids or 1x8, 1x6, or 1x4 strips with 2.4mm diameter exposed recording surface and a 1cm inter-electrode distance.

### ECoG data collection

Experimental recordings were conducted at the patient’s bedside without disruption of the clinical recordings. Either Synamps2 (Neuroscan, El Paso, TX, USA), or g.USBamps (GugerTec, Graz, Austria) sampled at 1000 Hz or 1200 Hz respectively were used for recording. ECoG potentials were recorded with respect to a reference electrode placed on the subject’s scalp. All stimulus presentation, real-time signal processing, and BCI feedback were conducted using the BCI2000 software suite [28].

### Cortical reconstructions and anatomical labeling

Cortical reconstructions were performed using previously published methods [29,30]. In brief, the reconstructions were generated as follows: Preoperative MRI was coregistered with postoperative CT imagery using the Statistical Parametric Mapping software package [31]. Reconstructions of the pial surface were then generated from the preoperative MRI using Freesurfer (freely available for download at http://surfer.nmr.mgh.harvard.edu/) and custom Matlab (The Mathworks, Natick, MA) code. Electrode positions were then estimated in the postoperative CT and projected onto the reconstructed pial surface following previously described methods [30].

Cortical surfaces and corresponding electrode locations were normalized to the Talairach brain using Freesurfer. For subjects with coverage of the left hemisphere, electrode positions were transposed to the right hemisphere for cross-comparison with other subjects. Anatomical labels corresponding to cortical regions were estimated using the human motor area template, a composite atlas based on the meta-analysis of 126 motor-based fMRI studies [32].

### Motor screening

Before subjects participated in any BCI trials, they first completed an initial screening task for the purposes of identification of a single electrode that was to be used for online BCI control. The task required the subject to perform overt and/or imagined movements in response to a visual prompt. Depending on each subject’s coverage, they performed gross hand motor movements (of the hand contralateral to the implant site), mouth motor movements, or both. Visual cues were presented for 3 sec followed by a 3 sec inter-trial interval. This sequence was repeated between 10 and 30 times. Band-limited power in the high-gamma HG range was estimated using a 4^th^-order Butterworth filter and the Hilbert transform. A single HG activation value was calculated on a per-electrode, per-trial basis by averaging all of the samples within that trial. The same process was conducted for all of the rest epochs. Electrodes that demonstrated statistically significant changes in HG activity from the rest period to the activity period for one of the two movement types were considered candidate electrodes for BCI control. In the case where there were multiple candidate electrodes, a single electrode was selected based on effect size, anatomical relevance, and researcher discretion. This electrode was then used in all subsequent recording sessions for BCI control and will be referred to in the remainder of the manuscript as CTL.

### The BCI task

**Fig 1:**
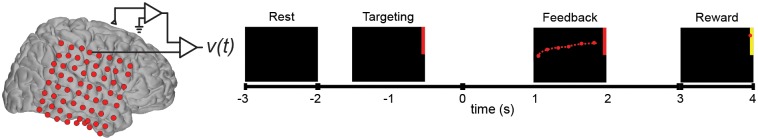
BCI task overview. Overview depicts the spatial scale of the ECoG grids, as well as the phases and timing of the BCI task. Subjects were presented with a target occupying either the upper (up target; depicted) or lower half (down target) of the right-most edge of the screen and had 3 sec to control the vertical position of the feedback cursor such that it ended the trial in the target area.

After initial screening tasks and electrode selection, the subjects were given as many opportunities as they wished to perform the 1-D, two-target right-justified box task. The task is discussed in our previous publications [8,29,33,34] and is reviewed in the following paragraphs. See Fig 1 for a depiction of the task.

The BCI task consists of four phases: rest, targeting, feedback, and reward, lasting, 1 second, 2 seconds, 3 seconds, and 1 second respectively.

During execution of the BCI task, the subject is presented with one of two targets, occupying either the top half or the bottom half of the right-most edge of the screen. After a fixed targeting interval of one sec, the cursor appears on the left edge of the monitor and travels to the right at a fixed horizontal velocity, such that the duration of the feedback period is fixed (3 sec all subjects but S6, who had a feedback period of 2 sec). The subject controls the vertical velocity of the cursor by modulating HG activity at the previously selected controlling electrode (CTL); performance of motor imagery causes the cursor to travel up and remaining at rest causes the cursor to travel down. Their objective is to complete each trial with the cursor in the specified target area for that trial. HG activity recorded at CTL is mapped to vertical cursor velocity using a simple linear decoder that was trained in the first set of trials. Throughout the remainder of this manuscript, targets occupying the top half and bottom half of the screen are referred to as “up-targets” and “down-targets,” respectively.

### Evaluation of behavioral performance

Subjects’ individual performance levels were calculated as the fraction of completed trials wherein the subject successfully ended the trial in the target area. Though theoretical chance performance on this task is 0.5, the behavioral performance necessary to be significantly greater than chance was dependent on the number of trials performed and thus varied from subject to subject. Confidence intervals on chance performance were evaluated on an individual basis by characterizing the distribution of average task performance under the null hypothesis that success and failure were equally likely outcomes on any given trial. To synthesize chance performance data we drew N random samples from the binomial distribution where N was the number of trials conducted by each subject; the average of these samples was one sample of chance performance under the null hypothesis. The distribution of chance performance was characterized by repeating this process 1000 times.

### Data pre-processing

Data were first manually inspected for any channels or time periods that contained obvious non-physiologic artifact or substantial inter-ictal activity. For each subject, the data were re-referenced by subtracting the common average among all good channels. Signals were then notch-filtered to remove line noise using 4^th^-order Butterworth filters at 60 and 120 Hz. For the purposes of short time windowed covariance (STWC) analyses, time-variant spectral estimates were extracted by bandpass filtering the signals using 4^th^-order Butterworth filters and then taking the magnitude of the Hilbert transform to determine the envelope of spectral activity. These spectral estimates were derived for the canonical high-gamma range (HG; 70–150 Hz). To reduce high-frequency noise, these spectral estimates were then temporally smoothed using a 47 msec full width at half maximum Gaussian window (100 msec wide).

**Fig 2:**
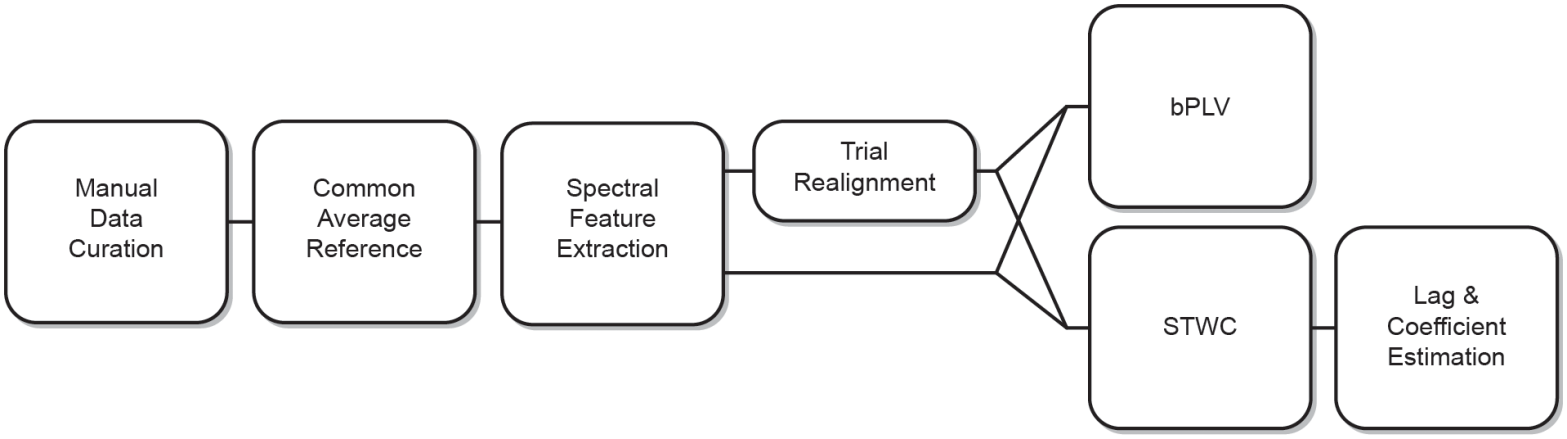
Post-hoc analysis workflow. Abbreviations: bi-phase locking value (bPLV), and short-time windowed covariance (STWC).

Finally, for computational tractability of remaining analyses, signals were then resampled to 400 Hz. Fig 2 gives an overview of the post-hoc analytical workflow.

### Trial realignment

Because of the potential for trial-to-trial variability in response time to the task, in addition to performing interaction analyses on trials aligned on cue presentation (cue-locked), we also performed these analyses on trials that were realigned based on initial onset of HG activity at CTL (response-locked). This allowed us to investigate both cue-driven and response-related interactions. Identification of this onset was performed as follows: First, the HG envelope for each trial was temporally smoothed using a 470 msec full width at half max (1 second wide) Gaussian window. Then a pre-onset baseline value was defined as the lowest value in the smoothed HG that occurred in the first second of the feedback period. The time at which this baseline value occurred was also noted. Next, the maximum value that occurred after the pre-onset baseline and before two seconds into the feedback period was also determined. The HG onset was defined as the first point after the pre-onset baseline when half the distance between the baseline and the maximum was crossed. All subsequent interaction analyses were performed on both cue-locked and response-locked trials.

It is noteworthy that this approach is only suitable when activity at the controlling electrode changes during feedback relative to rest. In previous work we demonstrated that subjects typically only modulate HG above baseline for up-targets [8], thus all interaction analyses computed on response-locked trials are based solely on activity changes during up-target trials.

### STWC analyses

Several methods exist to test for cross-frequency coupling: phase-amplitude coupling [35–37], bispectrum- and bicoherence-based measures [38,39] which involve both phase and amplitude, pure phase-based measures, i.e. the bi-phase locking measure [40] and amplitude-amplitude coupled measures, e.g. dynamic causal modeling [41] and short-time windowed covariance (STWC) [42]. While all these methods look for some form of cross-frequency interaction, they are each sensitive to different mechanisms that produce this cross-frequency coupling. While phase-amplitude coupling and bi-spectral/coherence have been extensively discussed in the literature, in this manuscript we focused on pure phase and pure amplitude based measures, which can be seen as testing for large ensemble to large ensemble interaction (amplitude-amplitude) and highly synchronized ensemble to highly synchronized ensemble (phase-phase), where the groups of neurons involved can be small.

We assessed transient temporal amplitude-amplitude correlations in HG activity between the CTL and remote electrodes using the normalized form of the STWC measure [42].

$$C\left(x,\;y,\;t,\;\tau ,\;\delta \right)=\frac{1}{\sigma_{x,t,\tau }\sigma_{y,t+\delta ,\tau }}\;\sum_{i=t-\frac{\tau }{2}}^{t+\frac{\tau }{2}}\frac{\left(x_{i}-\bar{x}\right)\left(y_{\delta +i}-\;{\bar{y}}_{\delta }\right)}{\left(\tau +1\right)}$$

Where *t* ∈[1,*T*] and *δ* ∈[−Δ,Δ] and *x* and *y* are the two signals being considered, *τ* is the window size over which the correlation is being calculated, *t* is the time (or sample) within the signal *x*, and *δ* is the lag of the window from *y* with respect to the window from *x*. $\bar{x}$ and ${\bar{y}}_{\delta }$ are the sample means and *σ*_*x*,*t*,*τ*_ and *δ*_*y*,*t*+*δ*,*τ*_ are the sample standard deviations from the two data windows. This method is specifically suited to teasing out amplitude-amplitude interactions in neural signals that are not only transient (e.g. event-driven), but also potentially occur at slightly different points in time in each of the two signals.

Individual STWC maps were calculated for each trial using a window width of 500 msec and a maximum lag of 300 msec. Average STWC maps were then generated separately for cue-locked and response-locked trials.

To isolate interactions relevant to task execution, we only evaluated interactions occurring within the first second of the feedback period (cue-locked trials) or ± 500 msec from HG onset at CTL (response-locked trials).

From each significant interaction, we extracted both a maximal STWC coefficient from the average STWC map as well as the corresponding lag at which this coefficient occurred. The former provides information regarding the relative strength of the interaction whereas the latter provides information as to the relative timing of the activity changes between the two areas.

### Bi-phase coupling

The bi-phase coupling value (bPLV) is a non-linear measure of cortical interaction. The bPLV can be computed from the time varying phase of the signal for a pair of frequencies as:

$$B_{XYZ}\left(t,\;f_{1},\;f_{2}\right)=\left|\frac{1}{N}\sum_{j=1}^{N}e^{i\left(\phi_{X}^{j}\left(t,\;f_{1}\right)+\phi_{Y}^{j}\left(t,\;\;f_{2}\right)+\phi_{Z}^{j}\left(t,\;f_{1}+f_{2}\right)\right)}\right|$$

Here $\phi_{x}^{j}\left(t,\;f_{1}\right)$ is the phase of signal *X* at frequency *f*_1_ and time *t* for the j^th^ trial, $\phi_{y}^{j}\left(t,\;f_{2}\right)$ is the phase of signal *Y* at frequency *f*_2_ and $\phi_{Z}^{j}\left(t,f_{1}+f_{2}\right)$ is the phase of the coupled signal *Z* [40]. The three signals (i.e. the sources X and Y and the target Z) can either be located in different positions, or in any combination be distributed across one to three electrodes. Here we choose a configuration, where the source signals, X and Y, reside in one location and the target is located at a different electrode. This configuration of the bPLV is similar to the one used in our earlier studies of bi-phase coupling in the motor system [43] and allows for an interpretation of causal directionality in the Granger sense, as here the phase at the target location is predicted by the phases of the source location, but not vice versa. Similar to our earlier studies, we compute trial-wise phase coupling, but we limit the source signals X and Y in this study to CTL and test for interactions to all other electrodes in the montage.

For a single pair of electrodes, we compute a frequency by frequency by time bPLV map, for interaction frequencies limited to coupling from 7–25 Hz to 70–100 Hz to a resulting target frequency ranging from 77 to 105 Hz. This range is motivated by our earlier studies [43], which found alpha/beta range coupling to high gamma frequencies during overt movement, as well as by the fact that this particular frequency range avoids the power line frequency at 60 Hz and its harmonics.

We use the continuous complex Morlet wavelet to compute a time varying phase for each frequency pair and the corresponding interaction frequency with a frequency resolution of 1 Hz. The resulting bPLV map per pair thus contains 589 time series, each 2200 samples long, covering the time from -3 s prior to the beginning of BCI control to approx. 2.5 s post control onset, when the trial ends. We compute these maps for all subjects across all channels in the montage over all trials.

We integrate the bPLV time series for each frequency pair from the onset of BCI control to 1 s post onset to test our hypothesis of a task specific increase in bPLV during execution of BCI control.

### Statistical testing and correction for multiple comparisons

Significance of STWC interactions was evaluated using a randomization approach on surrogate neural data. Using 100 iterations per channel pair, average STWC maps were calculated on trial-shuffled, phase-randomized neural signals. Phase randomization was used to destroy any temporal interaction between the two channels while preserving the individual power spectral characteristics of each channel. STWC maps were calculated as above and for each interaction, the maximal coefficient from the average maps for all channel pairs was retained to characterize the multiple comparison corrected distribution of maximal STWC coefficients one would expect to see under the null hypothesis of no interaction between electrodes [44]. Only STWC coefficients greater than 95% of this distribution were considered significant; reported p-values are the probability of seeing the observed STWC coefficient under the null hypothesis of no interaction.

Similarly, because of the large number of comparisons being made in the bPLV analysis, it is necessary to employ rigorous multiple comparison correction. Even after integrating the bPLV time series over a time interval, we are left with a large number of potential interactions and in the absence of a specific hypothesis about which frequencies and channel pairs should increase during BCI control, we use a statistical threshold to identify significant bPLV changes. While for individual bPLV values, an analytical expression for the null-distribution exists [40], we have no such description for the time integrated bPLV and thus must resort to non-parametric tests. We employed a similar maximum statistic approach as the one applied to STWC coefficients [44]. We use trial shuffling [20] to generate new samples of the time integrated bPLV, where we randomly shift trials between the controlling electrode and the target electrode. Here we assume that there exists no coupling on the time scale of full trails (which last >5s) and thus this method will generate an appropriate null-hypothesis. We generate 10,000 resamples per channel pair, but for each resample, after computing the integrated frequency by frequency map, we only retain the maximum value across all frequencies.

We then compute a p-value by comparing the original time integrated bPLV against the histogram of maximum values. This way we avoid having to control for multiple comparisons across the whole frequency by frequency map, which has a variable resolution, which would in case of a simple Bonferroni correction lead to a too conservative threshold.

Since channel pairs can be considered independent, we Bonferroni correct the resulting *p*-value from the maximum statistic by the total number of pairs examined.

We note that in the bPLV case, we could employ trial shuffling without phase randomization because there is no expectation of trial-to-trial consistency in absolute phase. Thus, by shuffling the trials we can destroy the structure of interactions sufficiently to generate the distribution of expected bPLV values under the null hypothesis of no interaction. Since STWC interactions are sensitive to amplitude, not phase, trial shuffling would be insufficient for characterization of the null distribution. Electrodes show a stereotyped amplitude response from trial to trial, which would remain consistent in spite of trial shuffling. Thus for characterization of the distribution of STWC interactions under the null hypothesis, we chose to employ both trial shuffling and phase randomization as the latter preserves the power-spectral nature of the signal while removing this stereotyped amplitude response.

## Results

### Behavioral performance

As is necessitated by our study inclusion criteria, all subjects performed above chance levels on the BCI task (*N* varies by subject, *binomial test*, *p* < 0.05). This is important to subsequent analyses as it serves to demonstrate that subjects had intentional control of the neural signal being used to control the BCI. S1 Fig shows that performance levels were above 95% chance performance confidence intervals for each subject; see S1 Table for additional detailed behavioral data.

### Response-locked STWC Interactions

**Fig 3:**
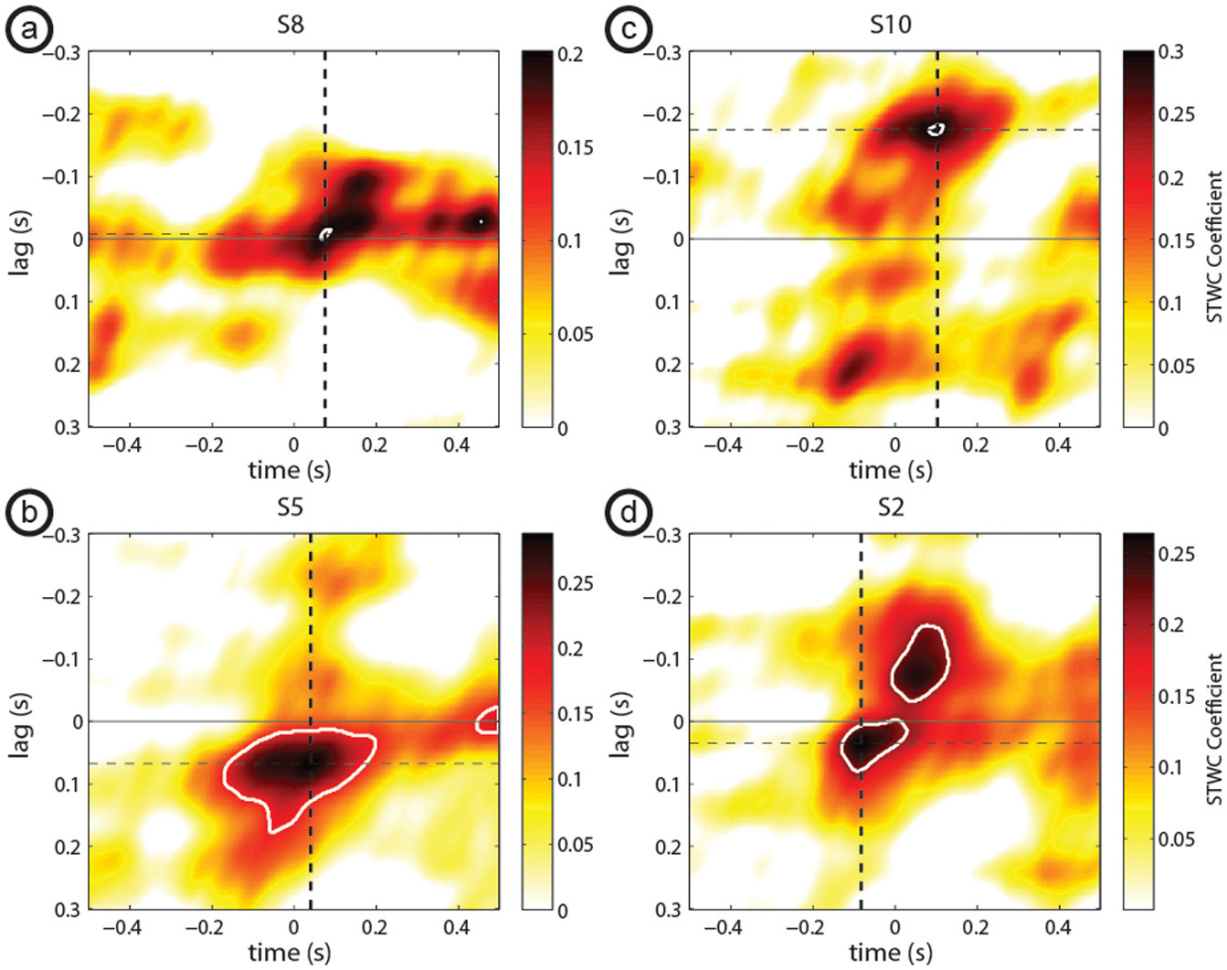
Response-locked STWC examples. Exemplar response-locked STWC maps from four subjects with significant STWC interactions showing a remote electrode (a) coactivated with, (b) leading, (c) lagging, or (d) both leading and lagging the CTL electrode. Significant interactions are circled in a white boundary. The solid black horizontal line depicts a lag of zero and dashed horizontal and vertical lines intersect at the peak STWC coefficient that was extracted and used in subsequent analyses.

When performing STWC interactions on response-locked trials, we identified 31 total electrodes, from a total of 9 of the 10 subjects that exhibited significant STWC interactions with the CTL electrode (*p* < 0.05; see randomization methods for detail). Exemplar interactions are shown in Fig 3. Information about all 31 response-locked interactions is given in S2 Table.

**Fig 4:**
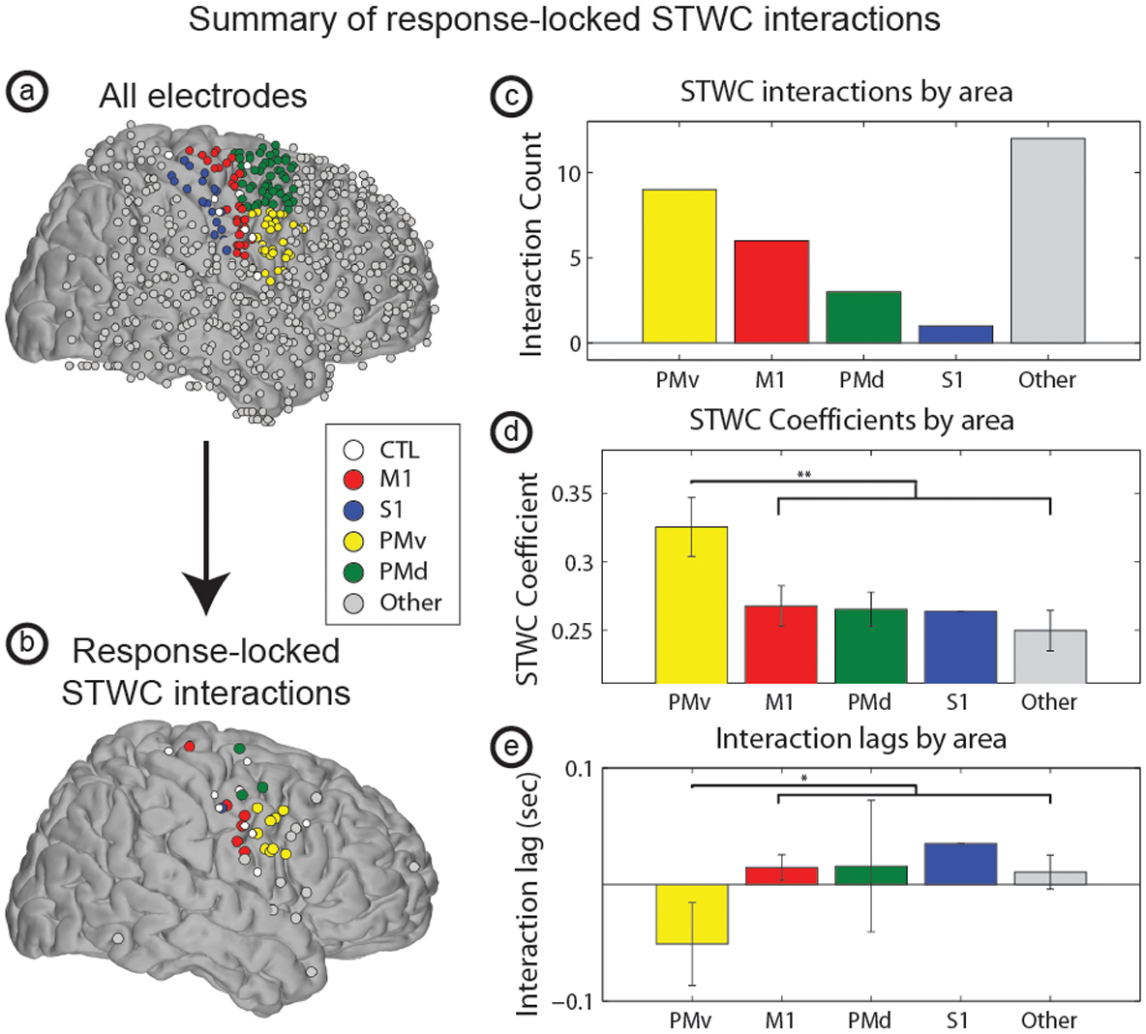
Spatial distribution of significant STWC interactions. Subplot (a) depicts the locations of all electrodes across all subjects whereas (b) shows both the CTL electrodes and the subset of all non-CTL electrodes that were involved in significant response-locked STWC interactions. Note that the majority of significant interactions were seen in the posterior portion of the frontal lobe. Subplot (c) shows the frequency of interactions in various cortical regions as defined by the human motor area template atlas. Subplots (d and e) show average STWC coefficients and lags (respectively) across those same regions. One star (‘*’) denotes p < 0.05 and two stars (‘**’) denote p < 0.01. Additional abbreviations: dorsal premotor cortex (PMd), primary somatosensory cortex (S1).

We note that though the electrodes considered in the STWC analyses come from a large number of cortical areas, as is depicted in Fig 4, and that there are a number of areas outside of traditional motor regions that are task-modulated during BCI, the areas interacting with the control electrode are almost exclusively contained to the posterior portion of the frontal lobe. Nine of the 31 electrodes interacting with CTL were found in ventral premotor cortex (PMv), meaning that 29% of observed significant interactions occurred within PMv, though less than 5% of all electrodes considered were over that area. Further, the interactions between this area and CTL showed larger overall STWC coefficients (*N*_*1*_ = 9, *N*_*2*_ = 22, two-sample *t*-test, *p* = 0.0017) and more of a tendency to lead CTL (*N*_*1*_ = 9, *N*_*2*_ = 22, two-sample *t*-test, *p* = 0.027) than interactions involving other regions. We also identified significant interactions between CTL and M1, primary somatosensory cortex, dorsal premotor cortex, and a small number of extra-motor areas.

### Cue-locked STWC Interactions

Additionally, we performed STWC analyses on cue-locked trials. We identified 23 total electrodes, from a total of 7 of 10 subjects that exhibited significant interactions with the CTL electrode (p < 0.05; see randomization methods for detail). Information for each of these interactions can be found in S3 Table.

**Fig 5:**
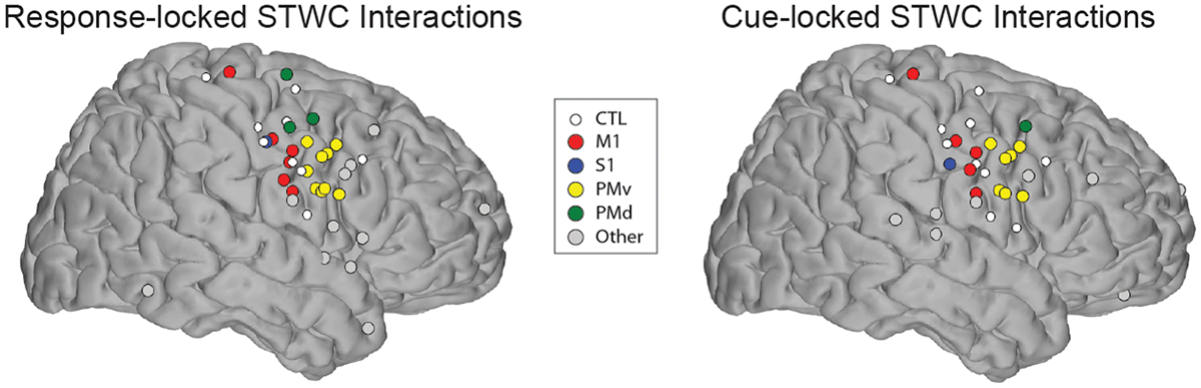
Spatial comparison of response-locked and cue-locked STWC interactions.

Again these electrodes were primarily located in the posterior portion of the frontal lobe; the spatial distribution of electrodes identified when applying STWC to unaligned trials is qualitatively similar to what was described for response-locked trials (Fig 5). Though slight, the decreased number of significant interactions on cue-locked trials relative to aligned trials (23 significant interactions as opposed to 31), combined with the fact that on average, significant STWC peaks calculated on cue-locked trials occurred 478 (± 36 SEM) msec after cue presentation suggests that the observed amplitude-amplitude interactions are involved more with the execution of motor imagery than the immediate response to the cue.

### Cue-locked bPLV Interactions

We observed significant cue-locked bPLV interactions in 8 of the 10 subjects. All significant interactions were ‘outgoing’ from CTL, meaning that the phases of an alpha and HG frequency at CTL were predictive of the phase at the sum of those two frequencies at a remote site.

**Fig 6:**
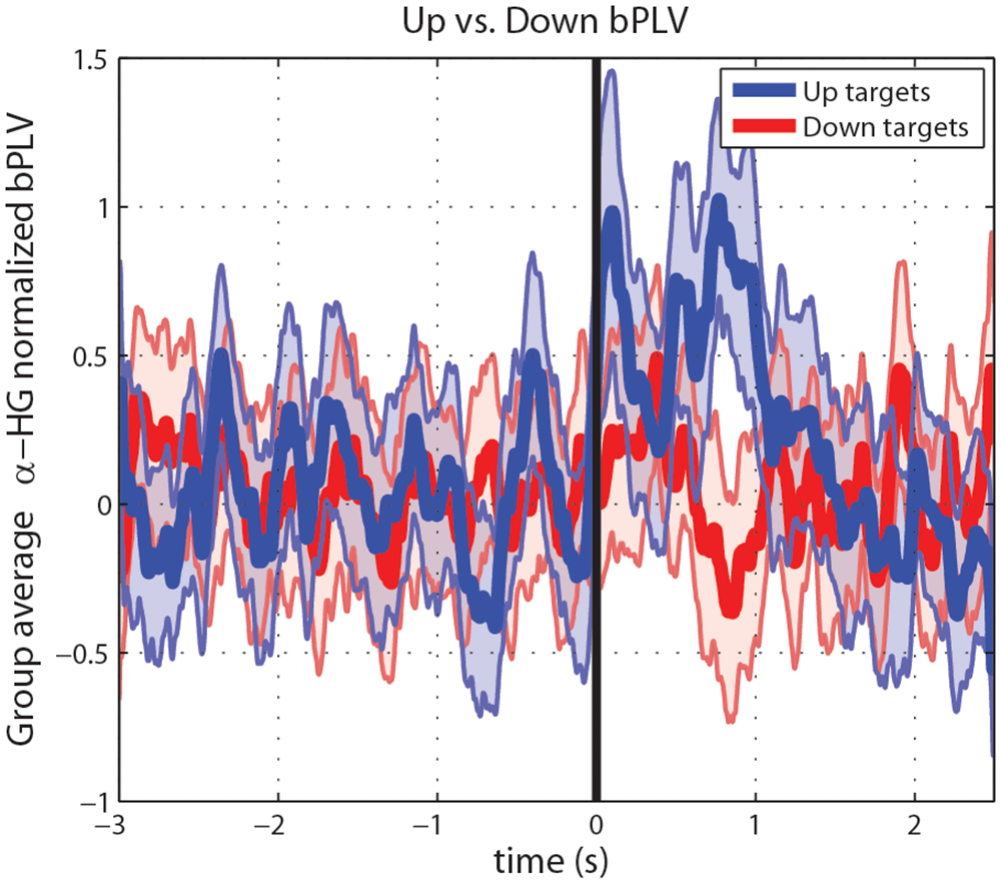
Group-average of significant bPLV interactions for up and down targets. bPLV values have been normalized on an individual subject basis using the bPLV timeseries from -3s < t < 0s; normalized bPLV, and thus the grand average is not necessarily bounded on the interval [0, 1]. Blue is the average for up-targets, red for down targets. The shaded areas show the 84% confidence interval (1 standard deviation) of the group average.

**Fig 7:**
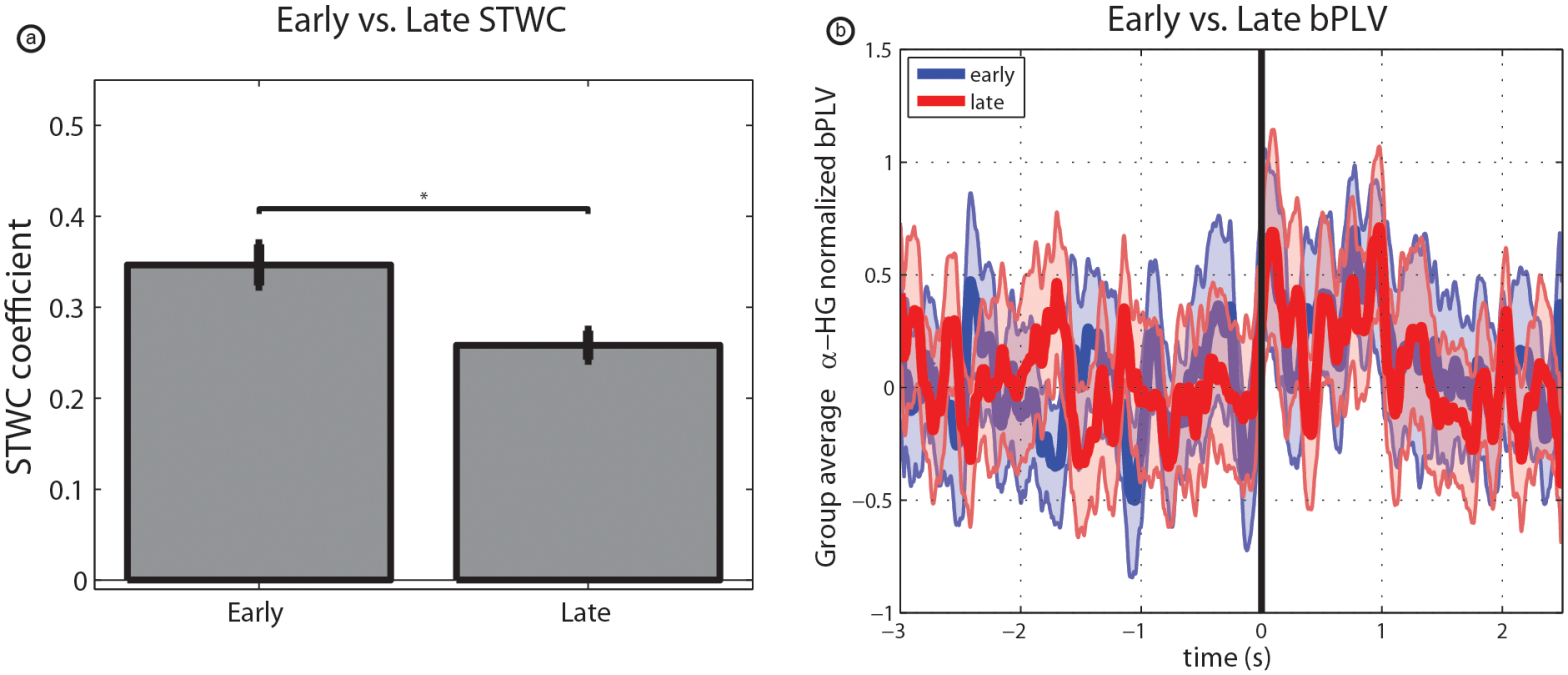
Changes in interactions over time. (a) Depicts change in peak response-locked STWC coefficients from early to late trials across all significant interactions. (b) Demonstrates that there was no significant change in grand-average normalized bPLV from early to late trials. One star (‘*’) denotes p < 0.05.

Since the bPLV measures are dependent on the number of trials from which they are computed (see [40] for a detailed discussion), measurements in individual subjects cannot be readily compared to each other. In order to form a grand average across subjects, we normalize individual bPLV timerseries to pseudo-zscores by computing the mean and standard deviation of the bPLV over the time interval before BCI control. We then subtract this mean from the bPLV timeseries and divide by the standard deviation. This procedure scales individual bPLV to a signal-to-noise ratio (SNR), which now can be compared across subjects as a grand average. This methodology is employed for both Figs 6 and 7.

Results of the grand average of the bPLV for individually significant alpha-HG interactions based on cue-locked trials are shown in Fig 6. Since bPLV significance for individuals was computed as an integrated value over a time interval, and the bPLV measures phase synchrony on a time scale of <10 ms, the individual time series over a scale of more than 5s can appear noisy and highly variable over time. However, on the group level, a stable trend emerges: an overall increase in bPLV between 0.5 s and 1 s post task onset, when comparing up vs. down targets, or up targets vs. baseline.

### Response-locked bPLV Interactions

We applied the same analysis (see methods) to response-locked trials, but under these conditions we observed no significant bi-phase coupling. Additionally, we evaluated linear phase-phase interactions from 1 to 200 Hz using the standard phase-locking value, and observed no robust trends across subjects in within-frequency coupling.

### Changes in interactions across skill acquisition

In order to begin to understand whether either of these interactions are indicative of the skill acquisition process, or are present during task execution in general, we re-evaluated all significant STWC and bPLV interactions using subsets of the trials. Comparing interactions from early trials (the first half of trials executed by a subject) and late trials (the second half of trials executed by a subject) we saw no statistical difference in bPLV interactions between remote electrodes and the CTL electrode (see Fig 7). This is in contrast with STWC interactions where we saw a significant decrease in median (per subject) interaction strength (paired *t*-test, N = 9, p = 0.016) from early to late trials.

One potential criticism of this finding is that though STWC is normalized for differences in amplitude, like any correlation measure, it is sensitive to changes in signal-to-noise (SNR). As we observed in our previous report [8], a number of cortical areas exhibit significant changes in task-driven HG activity that may—assuming a stationary noise floor—impact STWC strength over the course of skill acquisition. To control for this possibility we calculated SNR for all electrodes over the early and late trial periods, and repeated the above analyses excluding all interactions that involved electrodes with a significant decrease in SNR from early to late trials. In this case we still observed a significant decrease in median STWC strength (N = 8, p = 0.001) from early to late trials.

### Comparison of spatial distribution of STWC and bPLV interactions

**Fig 8:**
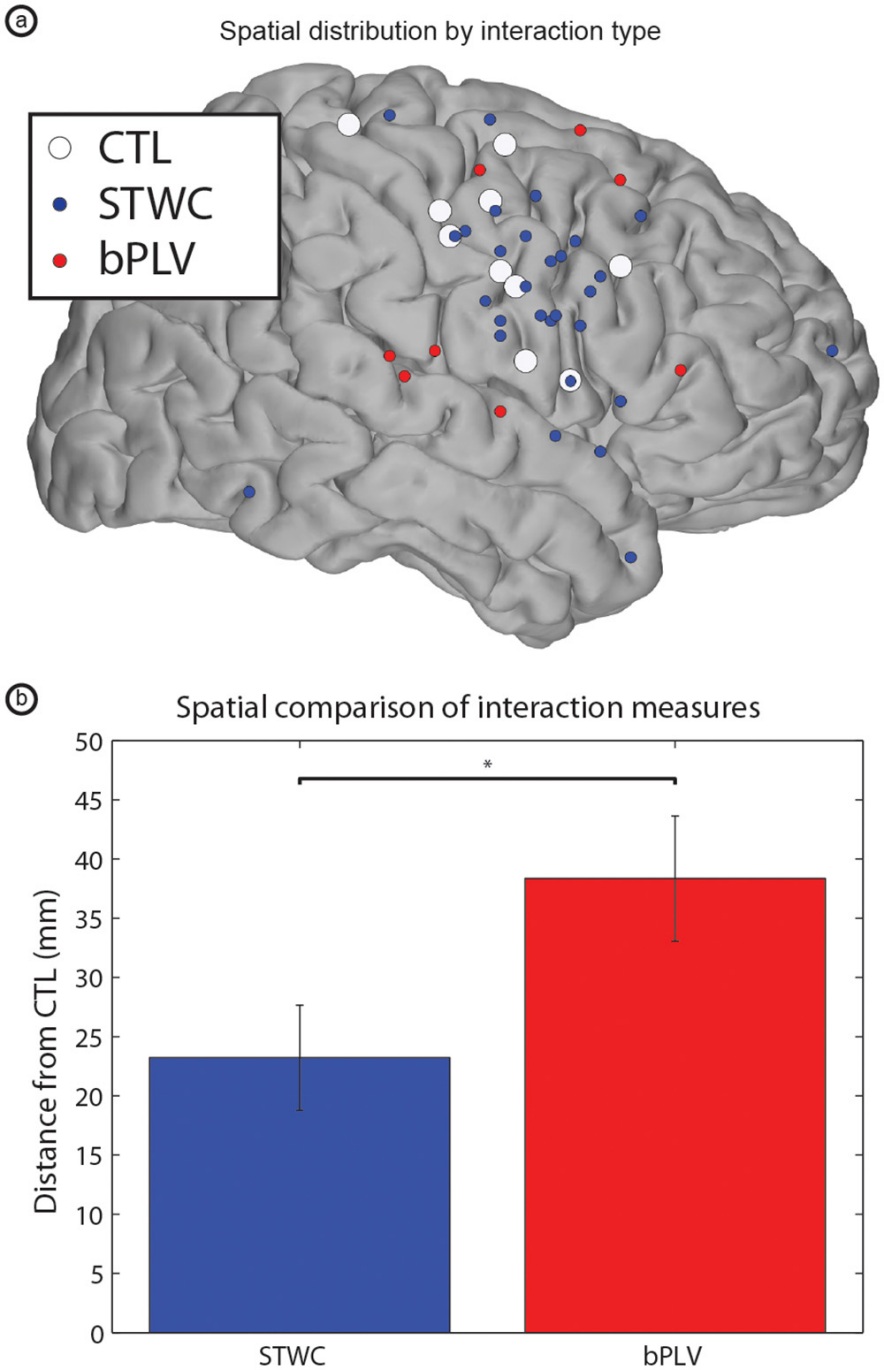
Comparison of spatial distribution of significant STWC and bPLV interactions. (a) Shows all electrodes involved in significant STWC or bPLV interactions across all subjects. Note the spatial localization of electrodes participating in STWC interactions to the posterior portion of the frontal lobe, and the slightly broader distribution of bPLV electrodes. (b) Provides quantification of this effect, comparing median STWC-CTL distances (per subject) with bPLV distances. One star (‘*’) denotes p < 0.05.

A number of electrophysiological studies suggest that the very nature of amplitude-amplitude and phase-phase interactions are different [26,45]. Though both may be indicative of information flow between cortical areas, the spatial and/or temporal scales over which these interactions take place may be quite distinct. With respect to spatial extent, we found this to be the case. Though we considered interactions from all cortical areas with electrode coverage, we primarily found significant STWC interactions close to primary motor cortex, often in PMv, or other nearby cortical regions. bPLV interactions, on the other hand, were much more spatially distributed, extending to dorsal premotor cortex, the superior temporal gyrus and prefrontal cortex. Considering the median STWC-to-CTL distance for each subject as a single observation to adjust for repeated measures within subjects, we found that the distance covered by STWC interactions was, on average, 23.22 mm, whereas the mean distance spanned by bPLV interactions was 38.35 mm, and that the distributions of these distances were significantly different (two-sample *t-test*, *N*_*1*_ = 8, *N*_*2*_ = 8, *p* = 0.04). Fig 8 provides additional detail as to the spatial distributions of these two interaction types.

## Discussion

In this manuscript we have demonstrated the presence of both HG amplitude-amplitude (STWC) and cross-frequency phase-phase (bPLV) interactions between cortical areas during BCI use. Coupling this with the fact that a number of cortical and subcortical regions have been shown to be active during the BCI task, execution of the neuroprosthetic skill is a coordinated effort involving multiple cortical areas. Additionally, we have demonstrated that cross-frequency phase-phase and within-frequency amplitude-amplitude interactions occur simultaneously during a cognitive task, but exhibit distinctly different spatial scales, which is indicative of at least two different modes of trans-cortical communication during BCI use. Lastly, we have demonstrated that the nature of these amplitude-amplitude interactions changes over the course of skill acquisition for a sub-group of the communicating regions. Our findings suggest multiple mechanisms of cortico-cortical communication that play differing roles in task execution.

Though our analyses included every implanted electrode for each subject, we found that the vast majority of significant STWC interactions occurred in or near the posterior portion of the frontal lobe and cover relatively short cortical distances (~2 cm). This not only implicates these regions in successful execution of a BCI driven by M1-derived control signals, but speaks to the relative temporal consistency of interactions between these areas and the controlling area and the possibility that these regions are responsible for similar facets of task execution. Given the tendency of neural circuits to optimize with BCI training [46], this may in part explain the observed decrease in STWC over the course of skill acquisition. As all subjects tended to develop proficiency with the task over time, it is difficult to interpret whether such decrease in interaction strength necessarily accompanies the learning process or is simply an artifact of repeated task execution. This remains an open question for future study, likely using more invasive approaches or active interventions to perturb the learning process. Along those same lines, it is interesting to note the relative absence of significant STWC interactions between CTL and cortical areas further upstream in the action portion of the perception-action cycle [47]. It is noteworthy that the pre-frontal cortex, which we have previously demonstrated to be active during BCI task execution [8], participated in relatively few significant STWC interactions with CTL. There are a few potential explanations for this: because there are few direct prefrontal-to-M1 connections [48], there may be a lack of temporal consistency in interactions between these areas that would render them statistically insignificant in our model-free analytical approach. Additionally, it is very likely that the task-relevant information represented in prefrontal cortex is more related to goal-direction [49,50] and working memory [51–53] than direct BCI control, thus we would not necessarily expect to observe tight temporal correlations between prefrontal cortical areas and M1.

The question remains of what function STWC interactions are playing to assist in BCI task execution. The fact that we observed temporal structure in STWC interactions that both followed and that preceded activity in the control electrode speaks to a potential feedback role for these regions. Signals that followed the control electrode activity could carry information about just-completed task performance whereas activity that precedes control electrode performance could carry information about motor planning that might result in improved task performance, though these mechanistic interpretations remain speculative.

Of particular interest is our observation that these high-frequency STWC interactions generally covered shorter cortical distances than bPLV interactions. This is in agreement with recent findings that inter-area correlations in the low-pass filtered HG envelope are more predictive of local than distant structural connectivity [27] and consistent with previous hypotheses regarding phase-phase interactions as an appropriate means for long-distance information transfer [26,43].

Oscillatory cortical activity has been studied extensively at multiple spatial scales [26], and, as a field, we have resounding evidence demonstrating that specific cortical oscillations respond reliably during sensorimotor and cognitive events [54] and relate to underlying neuronal firing patterns [55]. One prevalent hypothesis about inter-area oscillatory coupling is that it facilitates communication between two regions [56,57]; however, when such coupling is expressly linear (e.g., classical PLV), it comes at the expense of independent computation occurring in those two regions at those frequencies; this may be problematic for distant cortical areas performing different functions. Biphase coupling, on the other hand, is a proposed mechanism for information transfer between separated regions.

Bi-phase coupling measures the interaction of arbitrary phases at two frequencies (i.e., *f1* and *f2*) of a source site as it manifests at a target site. Since there are no constraints on the source site phases, information can be transferred from the source to the target by phase modulation of *f1* by *f2* at the source, resulting in a related phase of *f1*+*f2* at the target. Examples of similar coupling motifs can be seen in phase-modulated radio transmission [58].

It is worth noting that bPLV, in the context of ECoG studies, is being used to characterize the oscillatory interactions in relatively large networks. No hypothetical structural network has yet been described that models these interactions. They are being used solely as a means to characterize the nature of oscillatory interactivity between cortical regions

When evaluating multi-site interactions in any type of population-scale neural recording, it is important to consider the possibility that observed interactions may be in part due to effects of volume conduction. In the case of observed bPLV interactions, because of the expressly non-linear nature of this interaction measure, and the linear nature of volume conduction, it is extremely unlikely that observed effects could be explained by volume conduction between recording sites [40]. With respect to STWC, because the analyses were constructed to seek HG-HG interactions, it is possible that the effect is explained in part by volume conduction. In this case, interactions would have occurred only at zero lag relative to each other, which did not occur.

Cortical hubs proposed by Buzsáki et al. [22] and evidenced by Keller et al. [27] are intrinsically tasked with selectively processing information that comes from multiple streams simultaneously, attending to relevant information and muting the rest. Low-frequency oscillatory synchrony is one proposed mechanism for this gating [57,59,60]. However, if two streams of information are being integrated that occur on intrinsically different timescales (e.g., response to a visual stimulus and internal motor imagery state) the mechanism of linear phase-phase coupling may be insufficient and cross-frequency coupling may be a viable alternative.

Another possibility is that the role of information generated in one region may be different from its role in a distant region. If narrow-band HG changes are representative of selective activation of a subnetwork within an area [45] then biphase coupling presents one potential mechanism for transferring information from one such network to another, either within or between cortical regions. This is conceptually similar to the hypothesized role of phase-amplitude coupling: that it serves to transfer information across spatial and temporal scales, from distributed low-frequency oscillatory networks to local high-frequency ones [45].

As was described above, bPLV can readily be interpreted as a measure of effective (i.e. directed) functional connectivity. This is because the phases of the two multiplicative frequencies are predictive of the phase at the sum of their frequencies, but not vice versa. Whether STWC can be interpreted as a measure of functional (i.e. undirected) or effective connectivity depends, in part, on the lag at which correlative relationships occur. Though the potential existence of a hidden third source, exerting influence over the two visible nodes means that any conclusions as to causal influence of one of the visible nodes on the other must be tempered, at the very least, significant STWC coefficients at non-zero lags are indicative of information flow of some kind. Our finding of significant STWC lag relationships indicates that we are not merely observing non-specific co-activation of neighboring neural populations. The fact that we observed PMv typically leading CTL in significant PMv-to-CTL interactions makes sense considering traditional models of pre-motor influence on M1, but we note that this finding was based on extraction of a single lag value from each interaction. In reality we expect that there is likely bi-directional information flow between these and other cortical regions during BCI task execution.

The presence of bPLV synchronization exclusively during cue-locked trials is potentially indicative of the role that this synchrony may play in distributed processing of the task demands and subsequent execution planning. It may be that cortical synchrony is necessary to develop attentional focus or to create the appropriate state associated with task execution. The precise timing of task performance does not appear to impact bPLV measures: when the data were realigned to response times, the effects of bPLV change with the task largely vanished. The increase in bPLV lasted only over the initial portion of the task, meaning that bPLV changes had largely returned to baseline by the time the task was completed. This further supports that the cortical synchronization indicated by bPLV changes represents coordinated information flow related to the anticipation and state related to task performance rather than execution of the task itself.

Though there is evidence for cross-frequency coupling in both cortical [35,37] and cortico-subcortical networks [61], we have only limited data relating these oscillatory phenomena to activity changes in underlying neuronal networks [62]. Regardless of whether or not the observed macro-scale synchronization is directly related to neuronal computation or simply an epiphenomenon, through a better understanding of the behavioral mechanisms that generate them we will be able to extend our interpretations of these interactions when they are observed.

Structural interconnectivity and functional interactivity between populations of cortical neurons are at the core of human cognition. These interactions are dynamic and render in various ways in population-level cortical signals. Thus, although we have simultaneously demonstrated two different interactions in the ECoG surface potential, studies such as this will benefit greatly from an increased understanding of the anatomical mechanisms and network architectures that underlie the various forms of interactions observed in electrophysiological recordings.

## Supporting Information

S1 Table Individual behavioral results. (DOCX)

S2 Table Response-locked STWC interactions. (DOCX)

S3 Table Cue-locked STWC interactions. (DOCX)

S1 Fig Behavioral performance. (DOCX)
