# Supplementary material for: Cortico-Cortical Interactions during Acquisition and Use of a Neuroprosthetic Skill
Source: PLoS Comput Biol. 2016 Aug 19;12(8):e1004931. doi: 10.1371/journal.pcbi.1004931 (PMC4991818; doi:10.1371/journal.pcbi.1004931)
Supplement: S1 Table — (DOCX) [file pcbi.1004931.s001.docx]

Table S1 - Individual behavioral results. All represents the total number of valid BCI trials performed; performance is the fraction of those trials that were successful. In the second section we have listed the total number valid electrodes recorded from each subject. Lastly, response time is the mean time, in msec, between the start of the BCI trial and an increase in HG activity at CTL.

|  | BCI Trials | | Electrodes | Response Time |
| --- | --- | --- | --- | --- |
| **SID** | **All** | **Performance** | ***N*** | **Mean ± SD msec** |
| S1 | 84 | 0.8 | 82 | 908 ± 471 |
| S2 | 108 | 0.78 | 85 | 753 ± 385 |
| S3 | 39 | 0.72 | 63 | 886 ± 500 |
| S4 | 97 | 0.74 | 44 | 743 ± 482 |
| S5 | 164 | 0.9 | 94 | 788 ± 469 |
| S6 | 120 | 0.63 | 63 | 821 ± 412 |
| S7 | 68 | 0.76 | 63 | 733 ± 521 |
| S8 | 110 | 0.66 | 56 | 816 ± 484 |
| S9 | 89 | 0.64 | 61 | 986 ± 480 |
| S10 | 50 | 0.78 | 82 | 596 ± 370 |
