# Supplementary material for: Cortico-Cortical Interactions during Acquisition and Use of a Neuroprosthetic Skill
Source: PLoS Comput Biol. 2016 Aug 19;12(8):e1004931. doi: 10.1371/journal.pcbi.1004931 (PMC4991818; doi:10.1371/journal.pcbi.1004931)
Supplement: S2 Table — (DOCX) [file pcbi.1004931.s002.docx]

Table S2 – Response-locked STWC interactions. Table depicting all significant response-locked STWC interactions. Electrodes outside of the HMAT atlas are labeled as n/a.

| **Subject ID** | **Channel** | **HMAT** | **Lag (sec)** | **STWC Coeff** | **Talairach X** | **Talairach Y** | **Talairach Z** |
| --- | --- | --- | --- | --- | --- | --- | --- |
| 1 | 31 | RM1 | -0.005 | 0.285651 | 60.93164 | -7.497 | 32.8189 |
| 1 | 57 | n/a | -0.03 | 0.306682 | 30.93164 | 59.503 | 16.8189 |
| 2 | 32 | RS1 | 0.035 | 0.263841 | 59.93164 | -15.497 | 39.8189 |
| 2 | 39 | RM1 | 0.0425 | 0.267363 | 63.93164 | -9.497 | 26.8189 |
| 2 | 40 | RM1 | -0.015 | 0.27964 | 58.93164 | -6.497 | 36.8189 |
| 2 | 47 | RPMv | -0.035 | 0.453063 | 62.93164 | 1.503 | 23.8189 |
| 2 | 48 | RPMv | 0.0075 | 0.40401 | 58.93164 | 3.503 | 34.8189 |
| 2 | 54 | n/a | 0.055 | 0.222853 | 62.93164 | 7.503 | 10.8189 |
| 2 | 55 | RPMv | -0.1925 | 0.249224 | 61.93164 | 9.503 | 21.8189 |
| 2 | 56 | n/a | -0.025 | 0.225475 | 57.93164 | 13.503 | 31.8189 |
| 2 | 61 | n/a | -0.0825 | 0.214565 | 59.93164 | 13.503 | -3.1811 |
| 2 | 62 | n/a | 0.0425 | 0.225464 | 60.93164 | 17.503 | 6.8189 |
| 3 | 6 | n/a | 0.0075 | 0.336953 | -60.7254 | 4.417 | -0.0294 |
| 4 | 1 | n/a | -0.0025 | 0.211572 | 49.93164 | 19.503 | -24.1811 |
| 4 | 28 | n/a | 0.0475 | 0.310599 | 64.93164 | -6.497 | 19.8189 |
| 4 | 43 | RPMv | 0.0175 | 0.285704 | 53.93164 | 8.503 | 38.8189 |
| 4 | 44 | RPMv | 0.0075 | 0.292225 | 56.93164 | -1.497 | 39.8189 |
| 4 | 45 | RM1 | 0.0275 | 0.309015 | 57.93164 | -13.497 | 40.8189 |
| 4 | 50 | n/a | 0.0725 | 0.213988 | 42.93164 | 21.503 | 43.8189 |
| 4 | 52 | RPMd | 0.0175 | 0.287911 | 49.93164 | 0.503 | 47.8189 |
| 5 | 15 | RPMv | 0.0675 | 0.287107 | 62.93164 | 3.503 | 22.8189 |
| 5 | 16 | RM1 | 0.045 | 0.263375 | 63.93164 | -6.497 | 22.8189 |
| 5 | 94 | RSMA | 0.06 | 0.183838 | -0.06836 | -8.497 | 62.8189 |
| 7 | 63 | LPMd | 0.1125 | 0.263483 | -33.7254 | -8.583 | 62.9706 |
| 8 | 32 | RM1 | -0.0075 | 0.201601 | 37.93164 | -28.497 | 63.8189 |
| 10 | 9 | n/a | -0.06 | 0.230655 | 58.93164 | -56.497 | -11.1811 |
| 10 | 62 | RPMd | -0.0825 | 0.245022 | 55.93164 | -7.497 | 44.8189 |
| 11 | 38 | RPMv | -0.175 | 0.300494 | 62.93164 | 4.503 | 23.8189 |
| 11 | 39 | RPMv | 0.04 | 0.334831 | 61.93164 | -1.497 | 29.8189 |
| 11 | 46 | n/a | 0.0425 | 0.315437 | 59.93164 | 11.503 | 28.8189 |
| 11 | 47 | RPMv | -0.1975 | 0.322825 | 56.93164 | 5.503 | 35.8189 |
