# Supplementary material for: Cortico-Cortical Interactions during Acquisition and Use of a Neuroprosthetic Skill
Source: PLoS Comput Biol. 2016 Aug 19;12(8):e1004931. doi: 10.1371/journal.pcbi.1004931 (PMC4991818; doi:10.1371/journal.pcbi.1004931)
Supplement: S3 Table — (DOCX) [file pcbi.1004931.s003.docx]

Table S3 – Cue-locked STWC interactions. Table depicting all significant cue-locked STWC interactions. Electrodes outside of the HMAT atlas are labeled as n/a

| **Subject ID** | **Channel** | **HMAT** | **Lag (sec)** | **STWC Coeff** | **Talairach X** | **Talairach Y** | **Talairach Z** |
| --- | --- | --- | --- | --- | --- | --- | --- |
| 1 | 15 | n/a | 0.075 | 0.303426 | 65.93164 | -20.497 | 15.8189 |
| 2 | 21 | n/a | -0.3 | 0.224846 | 65.93164 | -34.497 | 13.8189 |
| 2 | 40 | RM1 | 0.02 | 0.209586 | 58.93164 | -6.497 | 36.8189 |
| 2 | 47 | RPMv | 0.0275 | 0.254644 | 62.93164 | 1.503 | 23.8189 |
| 2 | 48 | RPMv | -0.0225 | 0.242208 | 58.93164 | 3.503 | 34.8189 |
| 2 | 55 | RPMv | -0.0175 | 0.208279 | 61.93164 | 9.503 | 21.8189 |
| 3 | 9 | n/a | -0.19 | 0.339966 | -33.7254 | 44.417 | -12.0294 |
| 3 | 24 | LM1 | -0.03 | 0.395851 | -61.7254 | -8.583 | 30.9706 |
| 3 | 49 | n/a | 0.0425 | 0.335729 | -15.7254 | 63.417 | 23.9706 |
| 4 | 21 | n/a | -0.0775 | 0.211949 | 66.93164 | -20.497 | 8.8189 |
| 4 | 28 | n/a | 0.0475 | 0.293519 | 64.93164 | -6.497 | 19.8189 |
| 4 | 43 | RPMv | 0.0225 | 0.25431 | 53.93164 | 8.503 | 38.8189 |
| 4 | 44 | RPMv | -0.0125 | 0.271591 | 56.93164 | -1.497 | 39.8189 |
| 4 | 45 | RM1 | 0.0175 | 0.23496 | 57.93164 | -13.497 | 40.8189 |
| 4 | 51 | RPMd | 0.0575 | 0.227068 | 46.93164 | 10.503 | 45.8189 |
| 5 | 15 | RPMv | 0.075 | 0.222886 | 62.93164 | 3.503 | 22.8189 |
| 5 | 16 | RM1 | 0.0225 | 0.263872 | 63.93164 | -6.497 | 22.8189 |
| 5 | 20 | n/a | -0.025 | 0.188892 | 47.93164 | 33.503 | 27.8189 |
| 5 | 92 | RSMA | -0.1375 | 0.189673 | -0.06836 | -25.497 | 47.8189 |
| 8 | 32 | RM1 | -0.0075 | 0.218503 | 37.93164 | -28.497 | 63.8189 |
| 11 | 32 | RS1 | -0.1375 | 0.334892 | 62.93164 | -15.497 | 32.8189 |
| 11 | 46 | n/a | -0.085 | 0.307821 | 59.93164 | 11.503 | 28.8189 |
| 11 | 47 | RPMv | -0.055 | 0.31085 | 56.93164 | 5.503 | 35.8189 |
