# Supplementary material for: Cortico-Cortical Interactions during Acquisition and Use of a Neuroprosthetic Skill
Source: PLoS Comput Biol. 2016 Aug 19;12(8):e1004931. doi: 10.1371/journal.pcbi.1004931 (PMC4991818; doi:10.1371/journal.pcbi.1004931)
Supplement: S1 Fig — (DOCX) [file pcbi.1004931.s004.docx]

**
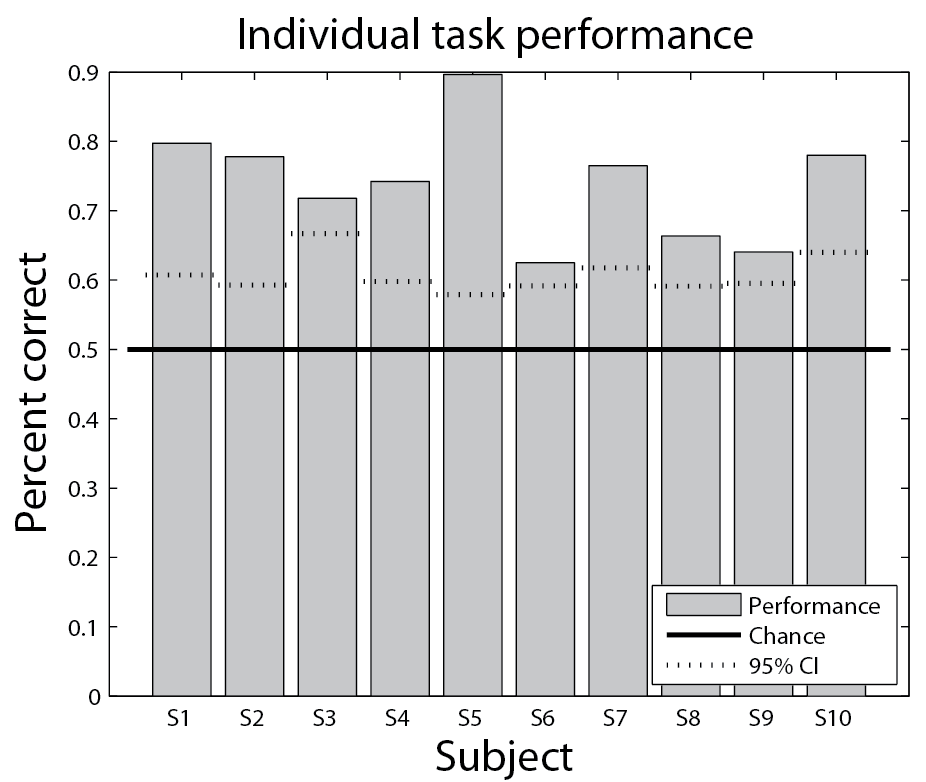
**

Figure S2 - Behavioral performance. Chance task performance denoted as black horizontal line. 95% Confidence intervals on chance performance differ by subject and are shown as a dashed line.
